# Choosing to Remain Childless? A Comparative Study of Fertility Intentions Among Women and Men in Italy and Britain

**DOI:** 10.1007/s10680-016-9404-2

**Published:** 2017-02-03

**Authors:** Francesca Fiori, Francesca Rinesi, Elspeth Graham

**Affiliations:** 1 ESRC Centre for Population Change, University of St Andrews (UK), Ladywell House, Ladywell Road, Edinburgh, EH12 7TF UK; 2 ISTAT, Italian National Institute of Statistics, Viale Liegi 13, 00198 Rome, Italy; 3 ESRC Centre for Population Change, School of Geography & Geosciences, University of St Andrews (UK), Irvine Building, St Andrews, KY16 9AL UK

**Keywords:** Childlessness intentions, Italy, Britain, Gender differences

## Abstract

Pathways to childlessness may differ not only between individuals but also at the population level. This paper investigates differences in childlessness by comparing two countries—Britain and Italy—where levels of childlessness are high in comparison with many other European countries, but which have distinct fertility trajectories and family regimes. Using data from two large, representative national samples of women and men of reproductive age in a co-residential partnership, it presents a rich analysis of the characteristics associated with intended childlessness, net of the aspects associated with being childless at interview. Although childlessness intentions are generally comparable between men and women of the same age, results show a link between socio-economic disadvantage and childlessness for British men as well as the importance of men’s employment for childbearing decisions in Italy. These findings support the view that pathways into childlessness are gendered and highlight the importance of partnership context in the understanding of fertility intentions. Then, the level of childlessness at interview is comparable across the two countries. However, a higher proportion of respondents in Italy is only provisionally childless, whereas a larger proportion of British respondents intends to remain childless. Framing these differences in fertility intentions within the wider context of family and fertility regimes allows insight into the extent to which observed levels of lifetime childlessness at the population level might result from a specific combination of intended childlessness, postponed decisions leading to involuntary childlessness, or constraints affecting abilities to achieve intentions at the individual level.

## Background

Since the 1970s, several European populations have experienced an increase in levels of childlessness, partly as a consequence of women progressively delaying procreation to later ages and the corresponding decrease in fecundity, and partly attributable to greater social acceptance of individual preferences for non-traditional, child-free life choices. Thus, pathways to childlessness may differ not only between individuals but also at the population level. This paper investigates differences in childlessness by comparing two countries—Britain and Italy—where levels of childlessness are high in comparison with many other European countries, but which have distinct fertility trajectories and family regimes. In doing so, it seeks to advance current analyses of childlessness by recognising that some childless individuals intend to remain childless, while others intend to have children in the future.

In the demographic literature, changing population patterns observed in European societies over the last few decades are typically framed by the narrative of the Second Demographic Transition (Lesthaeghe and Van de Kaa 1986), which attributes them to broader social transformations, especially the shift from materialism to post-materialism and consequent changes in the value system (Inglehart 1990). This would suggest that explanations for current levels of childlessness require greater attention to individuals’ aspirations and goals and their consequent life choices. With the emergence of more individualistic societies, or so the argument goes, more women will choose a child-free career-focused lifestyle. However, despite its emphasis on individualism, the Second Demographic Transition theory pays little attention to differences between, or within, populations beyond the observation that some populations are leaders and others laggards in this transformation (Graham 2014).

In contrast, Hakim’s (2000, 2003) *preference theory* offers greater insight into demographic diversity. It explains contemporary levels of childlessness by arguing that women are heterogeneous and have different preferences with regard to childbearing and lifestyle. She notes that the majority of women do not prioritise either employment (*work*-*centred* women) or family life (*home*-*centred* women) but are *adaptive* as their preferences respond to social pressures and policies. Thus, for the majority of women, choices about whether or not to have children can be seen not only as individual preferences but also as responses to their wider social, economic, and institutional circumstances.

In response to the excessive emphasis placed on ideational factors by Second Demographic Transition theory, other researchers have foregrounded institutional contexts and related low levels of fertility to social and cultural changes in gender roles that are affecting women’s lives. In countries where such changes have not been followed by adequate societal adjustment, women may be forced to make stark choices between work and family and, it has been argued, fertility has consequently fallen to very low levels (McDonald 1997, 2000). Most recently, Goldscheider et al. (2015) stressed the centrality of partnership and posited that structural changes in the relationship between men and women in the public and then in the private sphere play a crucial role in the understanding of current fertility trends and future trajectories. Differences in normative gender roles may therefore underpin fertility differences between Italy and Britain, including predominant pathways to childlessness.

Nevertheless, as similar levels of lifetime childlessness are observed within very different societies (and vice versa), explanations of remaining childless must go beyond differences in institutional contexts and recognise childlessness as the outcome of complex processes across various life spheres. Equally, researchers have pointed out the difficulties of framing childlessness entirely in terms of choice and preferences because childlessness rarely follows from a single decision (or non-decision). Some scholars have emphasised the distinction between being *childless* (or involuntarily childless, or childless by constraint) and *child*-*free* (or voluntarily childless, or childless by choice) (see, for instance, Tanturri and Mencarini 2008; Basten 2009). However, they have also recognised that the boundary between choice and constraint is often blurred and that childlessness may stem from a combination of both. Indeed, McAllister and Clarke (1998) devised a continuum of categories of childless people, distinguishing between those who were certain from a very early stage that they did not want any children, those who became certain that they did not want any children after experiencing some doubts in the past, those who accept childlessness, those who are ambivalent and, lastly, those who feel the decision was ‘taken for them’. This diversity is a reminder that preferences are not immutable and there are several pathways to lifetime childlessness, although such detailed distinctions are difficult to operationalise in a large-scale study. In order to contribute to a better understanding of childlessness, this study focuses on women and men in Italy and Britain who *intend* to remain childless, while noting that such an intention may change over time and is not always a matter of choice.

## Childlessness in Italy and Britain

In both countries, average completed family size by birth cohort has followed a decreasing trend, so that for women born in 1966, and reaching age 45 in 2011, completed family size was 1.91 children per woman in England and Wales (ONS 2013) and 1.50 in Italy (ISTAT 2014)—in both cases much smaller than among their mothers’ generation. Nevertheless, the explanations behind this decrease differ between the two countries. In Britain, the decrease in average family size has been accompanied by rising levels of childlessness, whereas decreasing fertility in Italy has been primarily the result of a dramatic drop in third- and higher-order births. However, although levels of childlessness have historically been lower in Italy, most recent estimates (ISTAT 2014) indicate that the proportion of childless women among the cohorts born after 1965 exceeds 20 % and is thus now comparable to levels in Britain.

In a European context, Britain stands out for its elevated proportion of larger families. Although a relatively large number of women remain childless, almost all of those who do become mothers have two or more children (Jefferies 2001). The polarisation of family size reflects the existence of heterogeneity in the tempo and quantum of childbearing across demographic and social groups that is perhaps more pronounced than elsewhere in Europe (Sigle-Rushton 2008). On the other hand, in Italy, the most prominent trait is that of a generalised delay of all life-course transitions among young people, including the end of education, entry into the labour market, leaving the parental family home, entry into co-residential partnership, and managing an independent household. This occurs within an institutional and cultural framework which has failed to adapt to changes in economic and social conditions, in particular to the increase in female education and employment (De Rose et al. 2008).

In sum, and despite different overall fertility levels and distinctive underlying patterns and historical trends of family formation and childbearing, levels of childlessness in the two countries among the youngest cohorts to complete their reproductive periods have converged. Yet, socio-cultural differences at the national level suggest that diverse processes may underlie this convergence.

Several scholars have argued that observed levels of childlessness are partly related to the onset and progression of recent family transformations across Europe (Sobotka 2004; Frejka 2008). Certainly, structural changes in women’s roles in the public sphere, especially in the labour market, have disrupted gender roles and challenged traditional notions of ‘the family’. In some contexts—and Italy is an example—greater economic responsibilities, combined with little relief from family roles, have led many women to compromise by delaying union formation and parenthood, although institutional influences, such as (the lack of) childcare provision and policies to facilitate combining work and childrearing, are also likely to be of importance. Other socio-cultural factors, and in particular a culture of childlessness, may also play a role. Arguably, Britain is characterised by a greater acceptance of child-free lifestyles and by a less marked disjuncture between public and private gender roles compared to Italy, so that high levels of childlessness coexist with overall higher fertility. Thus, although the prevalence of childlessness is similar in the two countries, its social acceptance (Sobotka and Testa 2008; Merz and Liefbroer 2012) and the extent to which it is intended or unintended (Hakim 2005; Kneale and Joshi 2008; Sobotka and Testa 2008) may differ greatly.

While theories of fertility variation have emphasised the gendered structure of society as a major element in the understanding of current childbearing patterns in different (national) contexts (Neyer et al. 2013), previous studies have shown that the inclination towards permanent childlessness at the individual level often differs by gender (Hakim 2005; Sobotka and Testa 2008). This suggests that childlessness intentions may be conditioned by different factors for women and men (Miettinen and Paajanen 2005; Sobotka and Testa 2008; Miettinen 2010), reflecting gendered life-course dynamics. Women experience the greatest difficulties in combining work and family life and often feel stronger pressures from both biological deadlines and social norms. Men’s childbearing, on the other hand, is more often conditioned upon their establishment in the labour market, although this association may be stronger where the breadwinner model of the family retains greater dominance.

## Research Design and Research Questions

The present study investigates the diversity of childlessness by examining differences between samples of (a) women and (b) men of reproductive age who are in a co-residential partnership in Italy and Britain. The empirical analyses exploit information on respondents’ current parity and their future fertility intentions, focusing on those who are childless at interview and do not intend to have children in the future. In particular, we follow the approach advocated by Rovi (1994), who argued that negative fertility intentions provide both a valid and reliable measure of childlessness since they are often found to be more stable over time than positive intentions and thus express permanence or commitment to a childless/child-free lifestyle (Westoff and Ryder 1977; Schoen et al. 1999; Noack and Østby 2002; Quesnel-Vallée and Morgan 2003). We restrict the analysis to those in a co-residential union in recognition of the negotiated nature of fertility intentions, which are likely to be more stable among those already living with a partner (Toulemon 1996). In addition, and in spite of a growing social acceptance of child-free life choices at least in Britain, it remains difficult for individuals to declare that they do not intend to parent. Thus, a negative response to a question on fertility intentions should suffice to distinguish individuals who are intentionally childless. Moreover, the ‘no’ answer is of interest in and of itself, as its study provides a way to understand better the current social milieu of individuals who reject the cultural mandate to parent.

Comparing samples of women and men from two countries with different fertility and family regimes also highlights the influence of socio-cultural differences at the population level, along with expected gender differences at the individual level within each country. In addition, individual-level analysis allows the identification of other significant determinants of intended childlessness in both countries.

Statistical analyses are designed to address the following research questions:

1. Are similar demographic and socio-economic characteristics associated with selection into childlessness in Italy and Britain?
2. Conditional upon being childless at interview, what are the main determinants of the intention to remain childless for women and for men in each country?

In the first question, the focus is on individuals’ current parity, i.e. on whether or not they are still childless at interview. The second question then examines the fertility intentions of those who are currently childless, focusing on those who intend to remain childless. Two groups of childless respondents are distinguished: those who are childless but do not intend to remain so (‘unintendedly’ or provisionally childless) and those who are childless and intend to remain so (‘intendedly’ childless). Both groups are heterogeneous. The first includes those who will go on to have children, those who postpone childbearing and may not realise their intentions due to reduced fecundity, and those who are not yet aware that they are infertile. The second group includes those who have for some time been certain that they do not want children, those who have recently become certain, and those who are sterile/infertile and are reconciled to childlessness, similar to the continuum identified by McAllister and Clarke (1998). In both cases, only a small minority, probably around 5 percentage, will be sterile/infertile.^1^ Further, the oldest women among the intendedly childless in our study are aged 35–39 and not yet at the end of their reproductive period, and all fulfil the normative prerequisite for family formation of living with a partner. Taken together, this implies that the intendedly childless group is dominated by those who have chosen to remain childless, both at the time of interview and for the foreseeable future.

In order to gain a better understanding of intended childlessness, our analyses are conducted in two stages. First, we investigate the profile of individuals who are childless at interview, as they may form a select group. Moreover, differences between Italy and Britain in those who remain childless at different ages may also be indicative of contextual influences on childlessness. Secondly, and taking account of selection into childlessness, we examine the determinants of intending to remain childless for women and men in both countries. Following the literature, we expect within-country gender differences in the factors associated with intended childlessness at the individual level. We also expect the determinants of intended childless for women and men in Britain to differ from those in Italy, where more traditional gender roles are still influential at a societal level.

## Data and Methods

### Data

Sample data are drawn from two recent nationally representative surveys. For Italy, we use the survey ‘Famiglia, Soggetti Sociali e Condizioni dell’infanzia’, 2009 edition, carried out by the Italian National Institute for Statistics (ISTAT). The survey collects a range of demographic and socio-economic information on a large sample of individuals from around 18 thousands households. Its retrospective design also allows the reconstruction of events over the life course, in particular with respect to education, employment, partnership, and fertility histories. For Britain, we use the first wave of the UK Household Longitudinal Study ‘Understanding Society’. The study started in 2009 and builds upon the long-standing British Household Panel Study (BHPS). The questionnaire covers a wide range of topics, such as family background, education, employment, finances, health and well-being, housing conditions, expectations, and attitudes. Compared to the BHPS, ‘Understanding Society’ is based on a much larger sample, interviewing participants aged 10 years and older from around 40 thousands households.

The two surveys were chosen from among other national surveys as they both contain a question on the fertility intentions of their respondents and cover the largest set of comparable demographic and socio-economic variables of interest. Some compromises, however, are necessary to ensure full comparability.

In relation to intended childlessness, the Italian survey asks respondents *‘Do you intend to have a child in the next three years?*’ and then, if the answer is negative, ‘*Do you intend to have a child in the future?’* The British survey only asks respondents *‘Do you think you will have any more/any children?’* without specifying a temporal reference. We therefore combine answers to the two questions for the Italian sample and, for both countries, consider the responses as indicative of lifetime intentions.

We limit the analysis of fertility intentions to childless respondents, defined as all women and men in the sample who never had natural, adopted, or step children (and whose partners also never had natural, adopted, or step children). Women who are pregnant at interview (or men whose partners are pregnant) are considered as childless with positive fertility intentions.^2^ Another difference between the two questionnaires relates to undefined fertility intentions,^3^ which are therefore excluded from our analyses.

We focus on respondents aged 25 and older, as fertility intentions of younger individuals are often less realistic and closer to ideal fertility (Régnier-Loilier 2006; Hayford 2009). We only include women up to age 39, since their fertility intentions might still be fulfilled. Fertility intentions at older ages tend to be more heavily constrained by the reality of declining fecundity as well as perceived risks, and possibly also normative sanctions on childbearing outside socially accepted ages (Ní Bhrolcháin et al. 2010). The upper age limit is set at 44 for men given that their fertility spans a longer period but that, on average, they tend to be only a few years older than their female partners.

As noted in the previous section, the main empirical analyses are further restricted to individuals living with a partner. The lack of a partner has been demonstrated to be an important predictor of childlessness (Heaton et al. 1999; Keizer et al. 2008; Tanturri and Mencarini 2008; Mynarska et al. 2015) as well as of the expression of negative fertility intentions (Miettinen and Paajanen 2005; Miettinen 2010; Sobotka and Testa 2008). Importantly, the decision to become a parent is usually made in the context of a partnership (Stein et al. 2014), where it results from complex interactions and negotiations between partners (Thomson 1997; Thomson et al. 1990). Further, fertility intentions expressed by partnered individuals better anticipate the couple’s future behaviour (Toulemon and Testa 2005), since they tend to reflect the conscious evaluation of preferences as well as contingencies and constraints.

The Italian sample comprises 3379 men aged 25–44 and 2745 women aged 25–39 living with a partner (of whom 660 and 571, respectively, are childless at the interview). The British sample consists of 4744 men and 3287 women (of whom 1244 and 896 are childless at interview).

### Variables

We study the intention to remain childless as a two-step process. We first study selection into childlessness at the time of interview, using as the dependent variable *Childless status at interview* (1: Respondent is still childless; 0: Respondent had a first child prior to the interview). Then, and limited to the subsamples of childless respondents, the dichotomous dependent variable is: *Intendedly childless* (1: Respondent intends to remain childless) vs. *Unintendedly childless* (0: Respondent intends to have children).

The choice of the explanatory variables, common to both the outcome and the selection equation, stems from the literature reviewed earlier in the paper and covers the main demographic and socio-economic factors that have been associated with differences in childlessness and expected reproductive behaviour in previous work (their percentage distribution is reported in the Appendix, Tables 3 and 4). The focus on respondents in a couple, together with data on the household, allows us to study both partners’ characteristics, thus offering some insight into the gendered nature of the couple relationship in the two countries and its influence on childlessness intentions.

The first set of variables accounts for the couple’s demographic characteristics: *Age* (25–29, 30–34, 35–39, and lastly 40–44 for men only), and *Age differences between partners* (More than 3 years younger, Same age, More than 3 years older); *Union typology* (Directly married, Married following cohabitation with the same partner, Cohabiting) and *Union duration* (Up to 2 years, 2–5 years, More than 5 years). Both partners’ *Perceived health status* (Good, Not good)^4^ is then included as a control for health issues potentially conditioning the reproductive plans of respondents.

Then, in order to account for the couple’s socio-economic status and the relative position of each partner, we considered the couple’s *Educational level* (Both high, Respondent high and spouse medium–low, Respondent medium–low and spouse high, Both medium–low) and each partner’s *Employment status* (In full-time employment, In part-time employment, Not in employment).^5^ Lastly, *Perceived economic situation* (Good, Not good) and *Housing tenure* (Ownership, Private renting, Other)^6^ are measured at the household level and further control for the socio-economic status of the individuals and their households.

### Methods

With respect to the analysis of intended childlessness, we face a potential problem of sample selection in that we can address our research questions only for respondents who were still childless at interview. This might be a non-randomly observed population. If unobserved factors affecting the intention to remain childless are correlated with the factors affecting the likelihood of being childless at interview, standard regression techniques deliver inconsistent estimations and lead to biased inferences about the outcome variable. We deal with potential problems of sample selection by applying a specification of the Heckman sample selection model (Heckman 1979) for dichotomous variables (van de Ven and van Praag 1981). The model consists of a system of two probit equations: a selection equation and the outcome of interest equation. The outcome equation measures respondents’ *Intention to remain childless* (Yes/No). Since childlessness intentions can only be observed if the respondent has no children at the time of interview, the selection equation then explicitly accounts for any selection bias by measuring *Childless status at interview*.

Formally, the sample selection model for dichotomous variables can be written as a system of equations for two latent variables:

1 $$y_{i}^{*} = x_{i}^{{\prime }} \beta + \theta S_{i} + u_{i}$$

where *I*_i_^*^ = 1 if the individual intends to have a child, and zero otherwise. In this context, *y*_i_^*^ represents the latent continuous variable, *β* is the vector of parameters to be estimated, *θ* is the coefficient associated with the endogenous dummy, and *u*_*i*_ is the residual term.

Similarly,

2 $$I_{i}^{*} = z_{i}^{{\prime }} \gamma + v_{i}$$

where *I*_i_^*^ = 1 if the respondent is childless, and I_i_^*^ = 0 if the respondent has already had a child. *I*_i_^*^ represents a latent continuous variable, *γ* represents a vector of parameters, and *v*_*i*_ represents the residual term. It should be noted that *y*_*i*_ can only be observed if *I*_i_^*^ = 1.

In standard regression models, *u*_*i*_ and *v*_*i*_ are assumed to be independent. But as we want to take into account potential sample selection, we have to consider the possible correlation between the two residual terms:

3 $$u_{i} = \lambda \varepsilon_{i} + \tau_{i}$$

4 $$v_{i} = \varepsilon_{i} + \varsigma_{i}$$

It is assumed that both *ε*_*i*_, *τ*_*i*_ and *ζ*_*i*_ are independently normally distributed, with mean 0 and variance 1. Finally, λ is a free parameter. If λ = 0, the correlation (*ρ*) between the two residuals terms equals 0 as well, and the hypothesis of sample selection can be rejected. In this case only, the ordinary regressions and the sample selection model will lead to the same results.

The Heckman-type selection models require that at least one explanatory variable that influences selection—but does not influence the subsequent process of interest—can be identified. To our knowledge, there are no other studies dealing simultaneously with childless status at interview and the intention to remain childless of childless respondents, and hence we cannot draw upon previous findings. Following an approach often encountered in the literature (see, among others, Philipov et al. 2006; Billari and Liefbroer 2007), our choice of instrumental variables is therefore informed by empirical trial: we select the variables referring to the family context in which respondents grew up (whether respondents have *Siblings*, *Divorced parents* and whether *At least one of their parents has tertiary education*) as preliminary analyses disregarding sample selection showed that they were significantly associated with childlessness at interview, but not with the intention to remain childless.

We estimate separate multivariate models predicting selection into childlessness, and then intended childlessness, for the two countries, and for men and women. However, to address our research questions, we require that coefficients across different models are comparable, in order to explicitly assess the existence of gender or country differences. We use the *suest* (seemingly unrelated estimations) procedure in Stata (Weesie 1999) which estimates the coefficients and standard errors of all models simultaneously. The coefficients do not differ from those obtained from separate estimations, but the standard errors are robust, which allows for their direct comparison.

## Results

### Selection into Childlessness in Italy and Britain

**Fig. 1:**
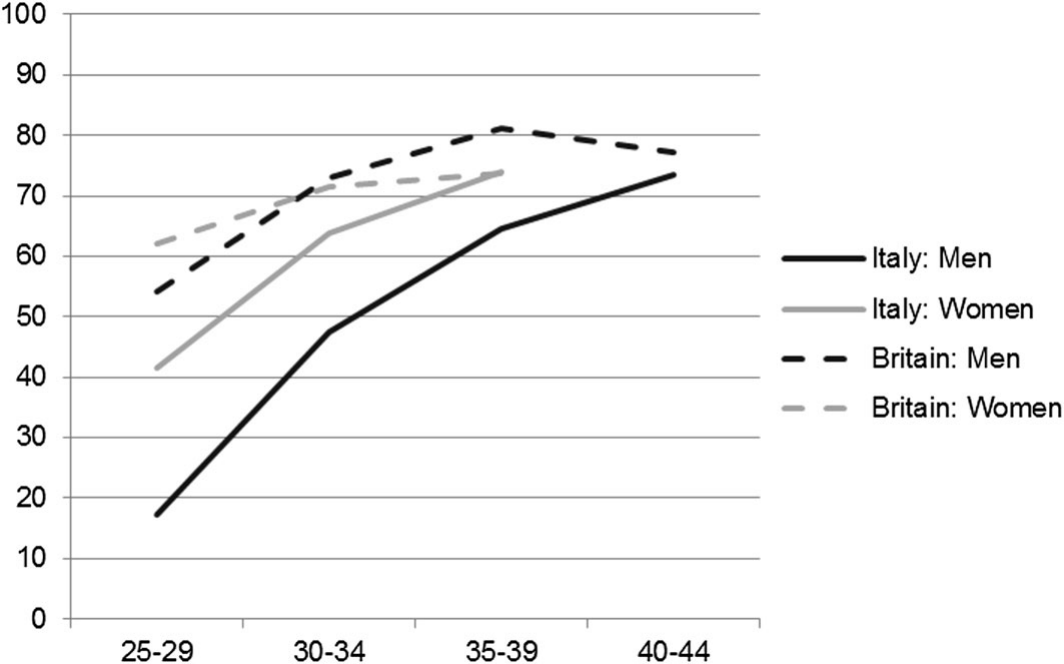
Proportion of all respondents living in a co-residential partnership. Men aged 25–44 and women aged 25–39, by country and age class. *Source*: Own elaborations on ISTAT ‘Famiglia, Soggetti Sociali e Condizioni dell’infanzia’, 2009, and UK Household Longitudinal Study ‘Understanding Society’, 2009

The multivariate analyses focus on individuals (men aged 25–44 and women aged 25–39) living in a co-residential partnership. Figure 1 shows their proportion (over the total population of the same age) in the two countries.

Among individuals aged 25–29, less than 20 % of Italian men, and 40 % of Italian women, are living in a co-residential union. Figures are much higher for British respondents of the same age, being around 60 % for both men and women. Among older individuals in our samples, however, the differences between Italy and Britain become negligible: in both countries just over 70 % of women aged 35–39, and approximately 75 % of men aged 40–44, are living with a partner (either married or cohabiting). The figures reflect the tendency of women to enter a co-residential partnership at earlier ages in both countries, although gender differences are more pronounced in Italy than in Britain. Most importantly, they confirm the well-established difference in the timing of union formation between Italy and Britain.

**Fig. 2:**
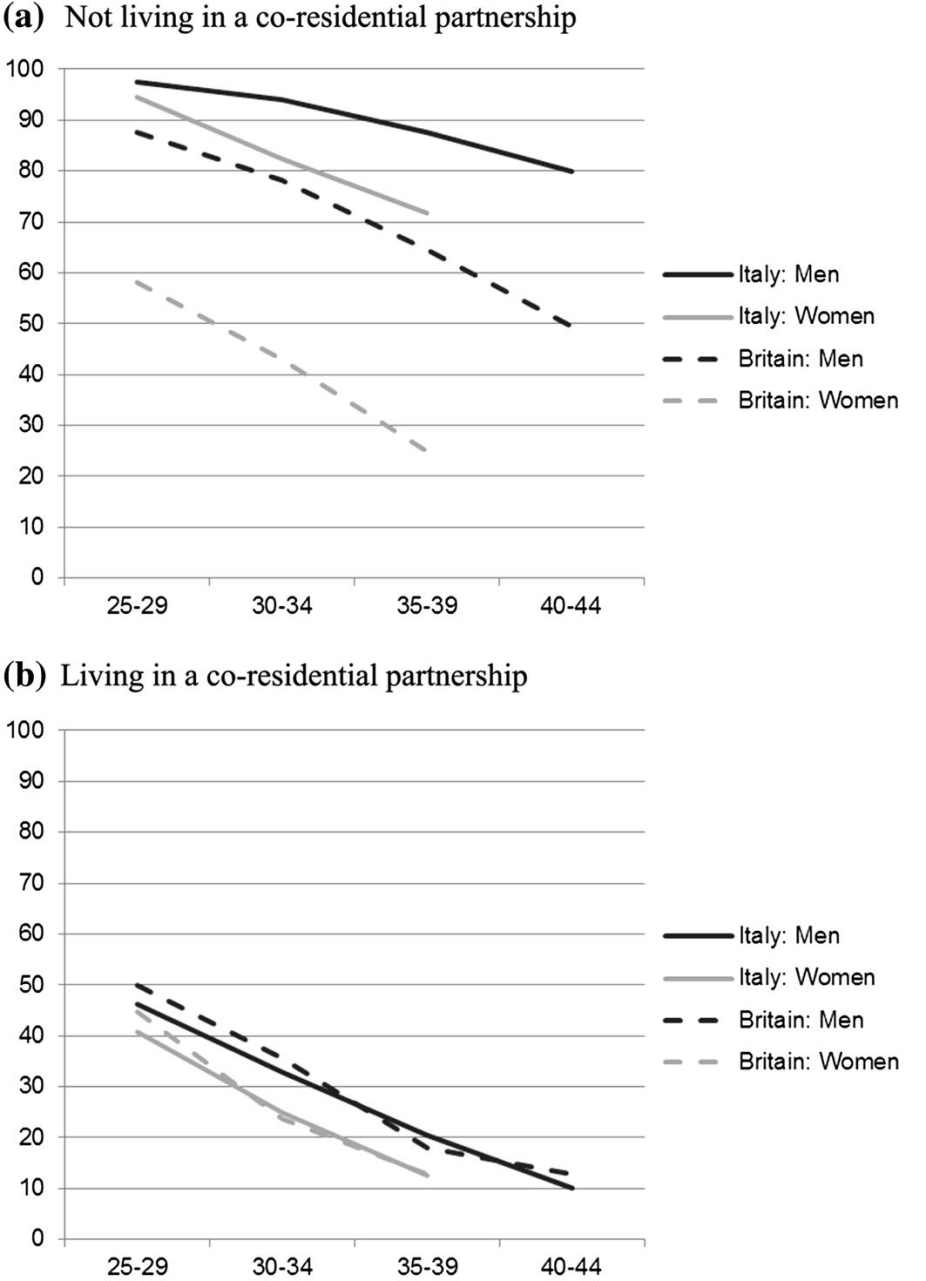
Proportion of all respondents who are childless at interview, by partnership status. Men aged 25–44 and women aged 25–39, by country and age class. *Source*: Own elaborations on ISTAT ‘Famiglia, Soggetti Sociali e Condizioni dell’infanzia’, 2009, and UK Household Longitudinal Study ‘Understanding Society’, 2009

Levels of childlessness at interview among respondents living with a partner are comparable between the two countries (Fig. 2). Overall, the proportion of childless respondents is around 19 % in Italy and slightly above 23 % in the British sample. The difference between the two countries in part reflects the different age structures of the samples since partnered respondents in the Italian sample are, on average, older than respondents in the British sample. On the other hand, in Italy, nearly all respondents in their late twenties who are not in a couple are childless. Figures for Britain are much lower, in particular for female respondents: just under 60 % of female respondents aged 25–29 and not living in a couple are childless at interview. Levels of childlessness among un-partnered respondents in Italy are consistently higher than in Britain across all age groups.

These figures confirm the greater importance of co-residential (marital) partnership as a prerequisite for childbearing in Italy compared to Britain and highlight how the later start of co-residential partnership in Italy directly translates into delayed entry into parenthood.

To answer our first research question, we now turn to the results of the first stage of the analysis and provide an overview of the factors associated with the likelihood of being childless at the time of interview, i.e. the selection equation.

Table 1 shows that not only are Italian respondents in a couple as likely as their British counterparts to be childless at all ages, but also that selection into childlessness is associated with similar socio-demographic determinants in both countries. All four models highlight a clear life-course dimension to childlessness, since respondents are less likely to be childless at older ages. Age differences between partners, however, are mostly not significant. Union duration is a strong predictor of childless status at interview, as the risk of still being childless at the time of interview decreases with longer union durations. A further demographic factor included in the model relates to union typology. Compared to respondents who married their partner directly without prior cohabitation, a current or past experience of informal cohabitation is generally associated with a higher likelihood of being childless. Childlessness is also more common when the female partner reports poor health status, but no significant effects are observed in relation to the male partner’s health status.

**Table 1:** Sample selection model—selection equation: probit model on being childless at interview. Coefficients and significance levels

Being childless at interview	Italy					Britain					Difference Italy versus Britain	
	(a)		(b)		(c)	(d)		(e)		(f)	(g)	(h)
	M		F		Diff M versus F	M		F		Diff M versus F	M	F
	β	Sig	β	Sig	Sig	β	Sig	β	Sig	Sig	Sig	Sig
*Demographic characteristics*												
Age classes												
25–29	0.30	*	0.27	**		0.26	***	0.53	***	**		*
30–34	Ref.		Ref.			Ref.		Ref.				
35–39	−0.09		−0.32	***	+	−0.44	***	−0.29	***		**	
40–44	−0.36	***				−0.56	***					
Age difference between partners												
Less than three years older	Ref.		Ref.			Ref.		Ref.				
Three years older or more	−0.02		−0.07			0.16	+	−0.09		*		
Union typology												
Directly married	Ref.		Ref.			Ref.		Ref.				
Married after cohabiting	0.23	*	0.16			0.02		0.17	*		+	
Cohabiting	0.35	***	0.38	***		0.46	***	0.49	***			
Union duration												
Up to 2 years	Ref.		Ref.			Ref.		Ref.				
2–5 years	−0.68	***	−0.70	***		−0.33	***	−0.44	***		*	
More than 5 years	−1.48	***	−1.44	***		−0.77	***	−1.02	***	*	***	*
Health (self-perceived)												
Good	Ref.		Ref.			Ref.		Ref.				
Bad	−0.01		0.36	*	+	0.11		0.31	**			
Health (self-perceived)—partner												
Good	Ref.		Ref.			Ref.		Ref.				
Bad	0.24	+	0.09			0.20	*	0.08				
*Socio*-*economic characteristics*												
Couple educational qualification												
Both High	0.14		0.01			0.55	***	0.57	***		**	***
Resp:high, partner: med–low	0.25		0.26	*		0.30	***	0.42	***			
Resp: med–low, partner: high	0.25	*	0.22			0.38	***	0.09		*		
Both Med–low	Ref.		Ref.			Ref.		Ref.				
Employment status												
Full-time employment	Ref.		Ref.			Ref.		Ref.				
Part-time employment	0.30	+	−0.41	***	***	0.03		−1.07	***	***		***
Not in employment	0.04		−0.74	***	***	0.15		−1.11	***	***		**
Employment status—partner												
Full-time empl	Ref.		Ref.			Ref.		Ref.				
Part-time employment	−0.33	***	0.18		*	−0.90	***	−0.26	+	***	***	+
Not in employment	−0.72	***	−0.07		***	−0.96	***	0.02		***	*	
Housing tenure												
Ownership	Ref.		Ref.			Ref.		Ref.				
Private rented	0.05		−0.03			0.23	**	0.26	***			*
Other	0.06		−0.06			−0.17	+	−0.06				
Economic situation (self-perceived)												
Not good	Ref.		Ref.			Ref.		Ref.				
Good	0.08		−0.03			0.39	***	0.44	***		**	***
*Family background*												
Siblings												
No	Ref.		Ref.			Ref.		Ref.				
Yes, one	−0.23	*	−0.19	+		−0.16	+	−0.21	*			
Yes, more than one	−0.25	*	−0.29	**		−0.25	**	−0.27	*			
Divorced parents												
No	Ref.		Ref.			Ref.		Ref.				
Yes	0.04		0.12			−0.13	+	−0.10				
Parents with tertiary education												
No	Ref.		Ref.			Ref.		Ref.				
At least one	−0.34	*	−0.02			0.14	*	0.15	*		**	
Constant	0.66	***	0.75	***		−0.12		−0.16			***	***

*** *p* < 0.001, ** *p* < 0.01, * *p* < 0.05, +*p* < 0.1

^a^Probit model, men, Italy

^b^Probit model, women, Italy

^c^Test of difference between coefficients for men and women in Italy. Significance level

^d^Probit model, men, Britain

^e^Probit model, women, Italy

^f^Test of difference between coefficients for men and women in Britain. Significance level

^g^Test of difference between coefficients for men in Britain and men in Italy. Significance level

^h^Test of difference between coefficients for women in Britain and women in Italy. Significance level

*Source*: Own elaborations on ISTAT ‘Famiglia, Soggetti Sociali e Condizioni dell’infanzia’, 2009, and UK Household Longitudinal Study ‘Understanding Society’, 2009

The second set of variables included in the model captures whether and how couple’s socio-economic status is associated with differences in the chances of being childless at interview, confirming the existence of greater social polarisation in Britain. In both countries, childlessness is least common when both partners have medium–low educational qualifications. On the other hand, it is most common in Britain when both partners have tertiary education, whereas in Italy it is the combination of a female partner with tertiary education and a male partner with low–medium education that shows the strongest association with childlessness at interview. Overall, educational differences are more pronounced in Britain. In both countries, and for both genders, respondents are more likely to be childless at interview if the female partner is in full-time employment, but household variables are significant predictors of childlessness only for Britain, with childlessness status for both British men and women being associated with perceived economic situation (i.e. respondents who say they are in a good economic situation are more likely, other things being equal, to be still childless at interview). Moreover, in Britain but not in Italy, compared to homeowners, male respondents in ‘other’ (socially rented) accommodation are marginally less likely to be childless, whereas higher childlessness levels are observed for both men and women in private renting.

Experiences in the family of origin also affect the chances of being childless at interview. In particular, having two or more siblings is associated with a lower likelihood of being childless at interview. For British men, parental divorce (perhaps reflecting the lower socio-economic status of the family of origin) is associated with lower risks of being childless at interview, whereas no significant effects are observed in Italy. Lastly, having a parent with tertiary education is positively associated with the likelihood of being childless for British respondents. The coefficient has the opposite direction for Italian men, who may be more financially secure if they come from a well-educated family.

Results from the selection equation allow us to draw a profile of respondents in the two countries who are childless at interview, and its simultaneous estimation with the equation on childlessness intentions ensures that the results presented in the next section are not biased by the selection process.

### Intending to Remain Childless

**Fig. 3:**
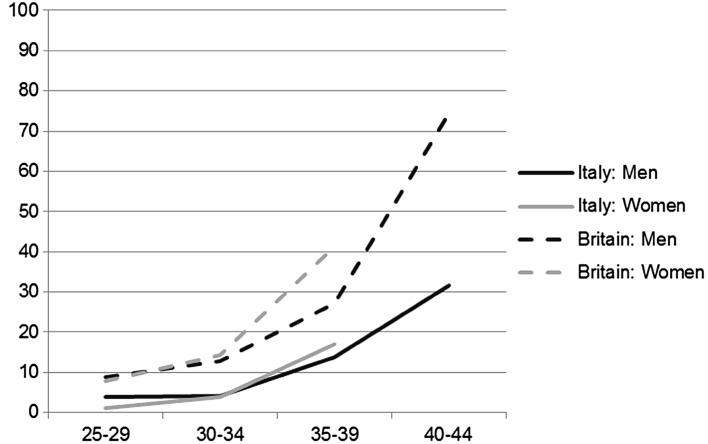
Proportion of all childless respondents living in a co-residential partnership at interview who intend to remain childless. Men aged 25–44 and women aged 25–39, by country and age class. *Source*: Own elaborations on ISTAT ‘Famiglia, Soggetti Sociali e Condizioni dell’infanzia’, 2009, and UK Household Longitudinal Study ‘Understanding Society’, 2009

The proportion of childless men and women living in a couple who say that they intend to remain childless is shown in Fig. 3. The breakdown by age reveals that levels of intended childlessness, as well as the differences between the two countries, increase with age. At younger ages, levels of intended childlessness are very similar for men and women within each country, although lower for Italy than for Britain. Thereafter, the trends diverge so that, within countries, the proportion of women in their late thirties who intend to remain childless is higher compared to men of the same age, and the increase is greater for Britain than for Italy.

The second stage of the analysis seeks to answer our second research question by comparing the main determinants of the intention to remain childless for women and men in each country. Table 2 summarises the results (β-coefficients and significance levels) from the estimation of the equation of interest, i.e. the probability of intending to remain childless at interview, adjusted for selection bias.

**Table 2:** Sample selection model—structural equation: probit model on intending to remain childless (conditioned upon being childless at interview). Coefficients and significance levels

Childless intentions	Italy					Britain					Difference Italy versus Britain	
	(a)		(b)		(c)	(d)		(e)		(f)	(g)	(h)
	M		F		Diff M versus F	M		F		Diff M versus F	M	F
	β	Sig	β	Sig	Sig	β	Sig	β	Sig	Sig	Sig	Sig
*Demographic characteristics*												
Age classes												
25–29	−0.05		−0.89	*		−0.18		−0.31				
30–34	Ref.		Ref.			Ref.		Ref.				
35–39	0.90	***	1.21	***		0.35		0.86	***	+		
40–44	1.63	***				1.65	***					
Age difference between partners												
Less than three years older	Ref.		Ref.			Ref.		Ref.				
Three years older or more	0.98	***	0.49	*		1.35	***	0.50	***	***		
Union typology												
Directly married	Ref.		Ref.			Ref.		Ref.				
Married after cohabiting	−0.12		−0.69	+		0.29		0.49	*			**
Cohabiting	0.31		0.12			0.85	**	0.77	**			
Union duration												
Up to 2 years	Ref.		Ref.			Ref.		Ref.				
2–5 years	−0.17		1.08			−0.08		0.33	+			
More than 5 years	0.89		1.96	+		0.20		0.19				
Health (self-perceived)												
Good	Ref.		Ref.			Ref.		Ref.				
Bad	−0.32		−0.09			0.10		0.44	*			
Health (self-perceived)—partner												
Good	Ref.		Ref.			Ref.		Ref.				
Bad	0.35		0.69	+		0.62	**	0.17				
*Socio*-*economic characteristics*												
Couple educational qualification												
Both High	−0.53		0.08			0.06		−0.30				
Resp: High, Partner: Med–low	−0.52		−0.23			0.46	*	−0.31		*	*	
Resp: Med–low, Partner: High	−0.51		−1.19			−0.07		−0.01				
Both Med–low	Ref.		Ref.			Ref.		Ref.				
Employment status												
Full-time employment	Ref.		Ref.			Ref.		Ref.				
Part-time employment	−0.04		0.20		**	0.71	*	−0.23		+		
Not in employment	0.84	**	0.18			−0.17		0.06			*	
Employment status—partner												
Full-time employment	Ref.		Ref.			Ref.		Ref.				
Part-time employment	−0.17		1.27	**		−0.26		0.01				*
Not in employment	−0.01		1.12	**		−0.35		0.27				+
Housing tenure												
Ownership	Ref.		Ref.			Ref.		Ref.				
Private rented	0.25		0.23			0.09		0.06				
Other	0.20		−0.60			0.29		−0.31		+		
Economic situation (self-perceived)												
Not good	Ref.		Ref.			Ref.		Ref.				
Good	0.03		0.65	*		0.47	*	0.32				
Inverse Mills ratio	−0.59		−1.07			0.59		0.26				
Constant	−1.79	**	−2.86	**		−2.93	***	−2.40	***			

*** *p* < 0.001, ** *p* < 0.01, * *p* < 0.05, + *p* < 0.1

^a^Probit model, men, Italy

^b^Probit model, women, Italy

^c^Test of difference between coefficients for men and women in Italy. Significance level

^d^Probit model, men, Britain

^e^Probit model, women, Italy

^f^Test of difference between coefficients for men and women in Britain. Significance level

^g^Test of difference between coefficients for men in Britain and men in Italy. Significance level

^h^Test of difference between coefficients for women in Britain and women in Italy. Significance level

*Source*: Own elaborations on ISTAT ‘Famiglia, Soggetti Sociali e Condizioni dell’infanzia’, 2009, and UK Household Longitudinal Study ‘Understanding Society’, 2009

#### Italy

In Italy, age is a significant predictor of the intention to remain childless for both women and men. Compared to the reference category of respondents aged 30–34, younger respondents are less likely to intend to remain childless, although the effect is significant only for women (*β* = −0.88, *p* < 0.05). Conversely, childlessness intentions are more common among older respondents. At age 35–39, the effect is more pronounced for women (*β* = 0.90, *p* < 0.001 and *β* = 1.21, *p* < 0.001, respectively, for men and women), although gender differences are not statistically significant. The age effect becomes stronger for men in their early forties (*β* = 1.63, *p* < 0.001). The fertility intentions of Italian respondents are also conditioned by their partners’ age. Respondents whose partners are more than 3 years older are more often intendedly childless, with the effect being larger for men living with older women than vice versa (*β* = 0.98, *p* < 0.001 and *β* = 0.48, *p* < 0.05, respectively, for men and women).

Fertility intentions expressed by childless men do not seem to respond to partnership variables. On the other hand, women are more likely to intend to remain childless if they have lived with their partner for more than 5 years (*β* = 1.96, *p* < 0.1), and less likely if they married following a period of cohabitation (*β* = −1.69, *p* < 0.1), although coefficients are only marginally significant. For both genders, however, it should be noted that simple probit models highlighted a positive and significant association between longer union durations and childlessness intentions. The association then loses significance and magnitude when selection into childlessness status is accounted for, thus suggesting that respondents are not more likely to remain childless the longer they live with their partner but rather that the association is likely to be endogenous.

Contrary to our expectations, respondents’ own health status is not associated with their fertility intentions, although women are more likely to intend to remain childless if their male partners report poor health (*β* = 0.68, *p* < 0.1). Nevertheless, for both genders, differences in intended childlessness are evident in relation to the employment status of the male partner. Men are more likely to intend to remain childless if they are not in employment (*β* = 0.84, *p* < 0.01); similarly, women express childlessness intentions significantly more often if their partners are not in employment (*β* = 1.12, *p* < 0.01) or only working part-time (*β* = 1.27, *p* < 0.01). At the same time, women are also more likely to intend to remain childless if they report a better household economic situation (*β* = 0.65, *p* < 0.05).

#### Britain

The fertility intentions of childless respondents in Britain are markedly influenced by their and their partners’ demographic characteristics. Again, age of both partners is an important predictor of fertility intentions. Older respondents are more likely to intend to remain childless compared to respondents aged 30–34. The effect is more pronounced for women in their late thirties compared to men in the same age group (*β* = 0.86, *p* < 0.001, for women), but, as in Italy, the age effect becomes significant for men in their early forties (*β* = 1.64, *p* < 0.001), reflecting their later (biological and social) fertility calendar. Age differences between partners also play an important role, as respondents living with older partners more often intend to remain childless; the effect of partner’s age is significantly stronger for men than for women (*β* = 1.35, *p* < 0.001 and *β* = 0.50, *p* < 0.001, respectively, for men and women).

Further, the type of partnership is associated with respondents’ fertility intentions. Those who are in a less traditional, possibly less committed, form of living arrangement (i.e. cohabitants) are more likely to intend to remain childless than respondents who married their partner directly (*β* = 0.85, *p* < 0.01 and *β* = 0.77, *p* < 0.01, respectively, for men and women). Married women with previous experience of cohabitation are also significantly more likely to express childlessness intentions (*β* = 0.49, *p* < 0.05).

Poorer health is another factor significantly associated with the intention to remain childless in Britain. However, in this case it is only the health status of the female partner that matters for both men (*β* = 0.62, *p* < 0.01) and women (*β* = 0.44, *p* < 0.05). Additionally, differences across socio-economic groups are evident only in relation to the fertility intentions of British men, who are more likely to intend to remain childless if they work part-time (*β* = 0.71, *p* < 0.05) and if they have medium–low education but live with a highly educated partner (*β* = 0.46, *p* < 0.05). Men reporting good economic conditions are also more likely to express childlessness intentions (*β* = 0.47, *p* < 0.05). Women’s fertility intentions, on the other hand, do not vary by socio-economic status.

These results reveal both differences and similarities in the demographic and socio-economic characteristics associated with selection into childlessness, in the determinants of the intention to remain childless in Italy and Britain, and between men and women in the two countries. Their wider contribution to the understanding of childlessness is discussed below.

## Discussion and Conclusions

Different family and gender role models are frequently referred to in the fertility literature, although the majority of past research has examined only women’s fertility. By investigating both gender differences and between-county differences in intended childlessness, this study contributes to the understanding of childlessness in Europe in several ways.

First, the idea that men might show a lower commitment to parenthood, although a recurrent theme in the literature, is only partially supported. Since our analyses are restricted to respondents living with a partner, we might suspect that men’s lower commitment to parenthood is concealed by their different propensity to start a cohabiting union. Indeed, our data show a lower proportion of men living in a co-residential partnership (especially in the Italian sample) and a higher proportion of men among childless respondents across all age groups. However, differences between genders are much smaller for those in a co-residential partnership and we find that childlessness intentions are generally comparable between partnered men and women of the same age, if not even lower among men. This suggests that childlessness among men reflects their later entry into partnership and their tendency to be, on average, older than their partners, rather than a lower inclination towards parenthood *per se*. Furthermore, whereas the ‘reality check’ (Sobotka and Testa 2008) that often leads to the downward revision of fertility intentions as women age (Berrington 2004) is well known, our study demonstrates a similar age effect among men, especially among those with an older partner. This highlights the importance of partnership context in the understanding of fertility intentions.

Secondly, our findings add further support to previous studies that have documented the link between socio-economic disadvantage and childlessness for British men (Berrington and Pattaro 2014; Jamieson et al. 2010), as well as the importance of men’s employment for childbearing decisions in Italy (Santarelli 2011; Vignoli et al. 2012). They are also consistent with the literature on low fertility which suggests that women’s increasing attachment to the labour market impacts on fertility indirectly through postponement of childbearing to later ages. In addition we find that, whereas the future childbearing intentions of childless women are not conditioned upon their current employment status, men’s employment status plays an important role in defining their intention to remain childless. In general, our study supports the view that pathways into childlessness are gendered—that being male or female shapes individuals’ trajectories (Keizer et al. 2008)—but we find that partner characteristics also matter.

Lastly, the differences between Italy and Britain in the gendered nature of partnerships reflect the importance of cultural context. We find that, although the level of childlessness at interview among respondents in a co-residential union is comparable across the two countries, a higher proportion of respondents in Italy is only provisionally childless (thus intending to have a child in the future), whereas a larger proportion of British respondents intends to remain childless. Framing these differences in fertility intentions within the wider context of family and fertility regimes allows insight into the extent to which observed levels of lifetime childlessness at the population level might result from a specific combination of intended childlessness, postponed decisions leading to involuntary childlessness, or constraints affecting abilities to achieve intentions at the individual level.

Men’s full-time participation in the labour force seems to be a necessary prerequisite for positive parenting intentions for both men and women in Italy, indicating the enduring influence of the male breadwinner family model in that country (De Rose et al. 2008). Combined with greater delays in the timing of union formation compared to Britain, and the consequent postponement of first births, this suggests that the failure to realise fertility intentions may be a major driver of low fertility in Italy. In contrast, a positive intention to remain childless may be having a greater impact on fertility levels in Britain where the individual preferences of selected socio-economic subgroups appear to play a more substantial role in influencing the likelihood of being, and intending to remain, childless. Thus, different mechanisms seem to be associated with the expression of negative fertility intentions in the two countries.

To our knowledge, this is the first comparative study on intended childlessness based on large, representative national samples, allowing us to conduct a rich analysis of the characteristics associated with intended childlessness in two European countries. The study may also be the first to explicitly model selection into childlessness and to examine the determinants of intended childlessness net of the aspects associated with being childless at interview. Nevertheless, the analyses underpinning our findings are not without certain limitations. Our choice of variables was constrained by the need to maintain comparability. We could not, therefore, include attitudinal measures (such as indicators of religiosity, gender roles, or family orientations) in our models. Further, compromises made because the wording of the question on fertility intentions is not identical in the two questionnaires may have resulted in negative fertility intentions being slightly over- or underestimated for certain subgroups, although not to the extent of changing our main findings. It is also worth noting that fertility intentions are subject to change over the life course, but are only measured in this study at one time point, albeit for respondents of different ages.

Although negative fertility intentions are generally considered relatively stable over time, longitudinal analyses of the extent to which intentions are revised during the life course could further extend understanding of the nature and drivers of childlessness. Some recent studies on Britain have investigated both this issue (Iacovou and Tavares 2011) and the realisation of fertility intentions (Berrington and Pattaro 2014). However, the stability of fertility intentions may differ between Italy and Britain, and more work is needed to investigate this possibility and its implications for our current findings. Future longitudinal research into the reproductive trajectories of childless individuals could also explore whether respondents who are provisionally, or intendedly, childless at interview achieve their fertility intentions by the end of their reproductive period, and whether the chances of realising intentions differ between Italy and Britain.

The socio-economic influences that tend to differentiate childless men and women in Britain and Italy may be less important than the cultural milieu in influencing the intention not to have children. To the extent that women and men who are intendedly childless are ‘choosing’ to remain childless, it appears that this lifestyle choice is more common in Britain. In contrast, lifetime childlessness in Italy is more likely to be involuntary and result from the postponement of fertility decision leading to a failure to realise the intention to have a child. We would expect the same constraints that shape the formation of negative fertility intentions to act as an obstacle to the realisation of intentions to have a child in Italy. In Britain, however, those who are provisionally childless may be more likely to realise their fertility intentions. As a consequence, we would expect a larger proportion of men and women in Italy compared to Britain to remain childless without intending that outcome.

When investigating childlessness, it is important to recognise that there are different pathways to lifetime childlessness. For the minority who cannot conceive for biological reasons, any choice is taken from them. For the majority, even those who choose not to have children, the choice is made within particular socio-cultural contexts. Whether childlessness is involuntary, intentional, or a result of the postponement of life choices matters because it will have different consequences for personal well-being and satisfaction later in life. Thus, different pathways into childlessness will have equally distinct implications for the efficacy of social and health policies designed to ease the economic and biological obstacles to parenting and raise or maintain national fertility rates.

## Appendix

Tables 3 and 4.

**Table 3:** Percentage distribution of the covariates included in the analyses. Women aged 25–39

	Italy			Britain		
	Respondent has children	Respondent is childless, but intends to have children	Respondent intends to remain childless	Respondent has children	Respondent is childless, but intends to have children	Respondent intends to remain childless
*Demographic characteristics*						
Age classes						
25–29	14.3	38.6	6.6	22.2	56.9	26.5
30–34	32.3	39.8	23.1	33.6	30.6	26.3
35–39	53.4	21.7	70.3	44.2	12.5	47.2
Age difference between partners						
Less than 3 years older	50.5	57.6	45.2	59.1	70.8	49.3
3 years older or more	49.4	42.5	54.8	40.9	29.2	50.7
Union typology						
Directly married	73.6	53.0	55.0	21.8	12.5	6.1
Married after cohabiting	17.2	19.9	9.3	52.9	37.1	38.4
Cohabiting	9.2	27.0	35.6	25.3	50.4	55.5
Union duration						
Up to 2 years	3.8	31.8	4.5	5.3	28.6	16.4
2–5 years	15.9	38.2	27.9	15.9	36.7	33.9
More than 5 years	80.3	30.0	67.6	78.8	34.8	49.7
Health (self-perceived)						
Good	92.7	93.6	77.9	87.9	92.8	82.3
Bad	7.3	6.4	22.1	12.1	7.2	17.7
Health (self-perceived)–Partner						
Good	91.4	93.7	77.5	87.1	92.8	87.0
Bad	8.6	6.3	22.5	12.9	7.2	13.0
*Socio*-*economic characteristics*						
Couple educational qualification						
Both High	7.5	12.6	6.9	26.8	50.1	36.2
Resp: High, Partner: Med–low	9.5	18.7	20.8	15.8	23.7	20.3
Resp: Med–low, Partner: High	3.8	4.1	2.8	12.2	8.4	11.0
Both Med–low	79.1	64.7	69.5	45.1	17.9	32.5
Employment status						
Full-time employment	31.9	61.7	68.0	33.2	81.7	76.4
Part-time employment	18.0	15.0	9.6	33.3	9.7	8.5
Not in employment	50.2	23.3	22.4	33.6	8.6	15.1
Partner’s employment status						
Full-time employment	88.7	89.9	71.8	82.7	90.0	85.7
Part-time employment	4.1	5.0	16.1	4.5	3.2	1.6
Not in employment	7.1	5.2	12.1	12.8	6.8	12.6
Housing tenure						
Ownership	61.9	63.6	70.1	67.0	60.4	67.2
Private rented	25.7	23.4	25.6	15.9	33.7	25.2
Other	12.3	13.0	4.3	17.1	5.9	7.5
Economic situation (self-perceived)						
Not good	36.3	29.3	27.2	45.5	23.5	24.7
Good	63.7	70.7	72.8	54.5	76.5	75.3
*N*	2173	531	40	2391	754	142

Source: Own elaborations on ISTAT ‘Famiglia, Soggetti Sociali e Condizioni dell’infanzia’, 2009, and UK Household Longitudinal Study ‘Understanding Society’, 2009

**Table 4:** Percentage distribution of the covariates included in the analyses. Men aged 25–44

	Italy			Britain		
	Respondent has children	Respondent is childless, but intends to have children	Respondent intends to remain childless	Respondent has children	Respondent is childless, but intends to have children	Respondent intends to remain childless
*Demographic characteristics*						
Age classes						
25–29	4.3	15.9	4.5	13.4	43.5	13.1
30–34	18.5	38.9	11.7	20.6	33.9	18.7
35–39	31.8	31.1	34.7	31.5	18.3	22.5
40–44	45.4	14.0	49.1	34.4	4.3	45.7
Age difference between partners						
Less than 3 years older	94.3	94.3	79.4	89.9	95.0	70.5
3 years older or more	5.7	5.7	20.7	10.1	5.0	29.5
Union typology						
Directly married	75.8	55.2	53.4	21.6	13.4	10.2
Married after cohabiting	15.6	19.3	14.3	52.2	33.5	32.3
Cohabiting	8.5	25.5	32.3	26.2	53.1	57.6
Union duration						
Up to 2 years	3.7	29.7	17.3	7.1	30.3	17.0
2–5 years	14.0	39.9	16.6	17.1	38.5	18.3
More than 5 years	82.3	30.4	66.1	75.8	31.1	64.7
Health (self-perceived)						
Good	91.2	94.2	91.9	87.0	92.2	86.5
Bad	8.8	5.8	8.1	13.0	7.8	13.5
Health (self-perceived)—partner						
Good	92.2	94.2	82.8	87.1	93.0	81.7
Bad	7.8	5.8	17.2	12.9	7.0	18.3
*Socio*-*economic characteristics*						
Couple educational qualification						
Both High	7.7	12.9	4.6	26.2	49.0	31.9
Resp: High, Partner: Med–low	3.8	4.1	6.3	12.8	10.0	18.5
Resp: Med–low, Partner: High	9.7	19.5	11.9	15.7	21.5	17.9
Both Med–low	78.8	63.5	77.2	45.3	19.5	31.8
Employment status						
Full-time empl	90.3	89.5	79.8	83.0	89.4	84.9
Part-time empl	3.8	5.4	7.7	4.8	3.4	5.2
Not in employment	5.9	5.2	12.5	12.2	7.2	10.0
Employment status—partner						
Full-time empl	33.0	60.6	66.8	36.8	78.7	71.2
Part-time empl	18.3	15.4	12.9	31.8	10.3	14.1
Not in employment	48.8	24.0	20.2	31.4	11.0	14.7
Housing tenure						
Ownership	63.3	62.6	54.8	67.8	58.8	71.5
Private rented	24.3	24.8	28.8	15.6	35.4	17.5
Other	12.4	12.6	16.4	16.6	5.9	11.0
Economic situation (self-perceived)						
Not good	36.7	28.9	30.9	45.9	28.3	26.0
Good	63.3	71.1	69.1	54.1	71.7	74.0
*N*	2720	571	89	3500	961	283

Source: Own elaborations on ISTAT ‘Famiglia, Soggetti Sociali e Condizioni dell’infanzia’, 2009, and UK Household Longitudinal Study ‘Understanding Society’, 2009

## Footnotes

1 In a study of the 1970 birth cohort in Britain, for example, Kneale and Joshi (2008) found that, of women who were childless at age 34, only 4.2 % were sterile/infertile.

2 This categorisation is imposed by the data, since the Italian questionnaire does not distinguish pregnant women as a separate category and records them as childless respondents intending to have a child. On the other hand, the British questionnaire explicitly identifies respondents who are pregnant (and their partners), who are not surveyed about their fertility intentions.

3 The British questionnaire codes respondents who answer ‘don’t know’ as a distinct category, whereas in the Italian questionnaire, they are grouped together with respondents who refuse to answer the question.

4 As our samples are made up of relatively young and healthy individuals, the variable only singles out the minority reporting poor or very poor health conditions.

5 A more refined categorisation is not used due to the small sample size—particularly for the study of fertility intentions of childless respondents—which implies we will incur convergence and estimation issues.

6 Within the British context, the category ‘Other’ mostly consists of individuals in rented social housing. Social renting, on the other hand, is not a common tenure in Italy, and the category ‘Other’ in this case includes mainly individual living rent-free in family-owned houses.
